# Fathers’ involvement in pregnancy and childbirth in Africa: an integrative systematic review

**DOI:** 10.1080/16549716.2024.2372906

**Published:** 2024-07-12

**Authors:** Samuel Nambile Cumber, Anna Williams, Helen Elden, Malin Bogren

**Affiliations:** a Institute of Health and Care Sciences, Sahlgrenska Academy, University of Gothenburg, Gothenburg, Sweden; b Data, Design + Writing, USA

**Keywords:** Africa, childbirth, gender norms, father involvement, integrative review, pregnancy

## Abstract

**Background:**

As notions of masculinity evolve globally, it is important to understand their dimensions within geographic regions and life contexts. African men’s involvement in their partners’pregnancy and childbirth has been explored to a limited extent in the peer-reviewed literature. This analysis provides a comprehensive examination of the existing literature on the diverse experiences of fathers across the African continent.

**Aim:**

This study aims to provide an overview of fathers' experience of involvement in their partners’ pregnancies andchildbirth in Africa.

**Methods:**

A systematic integrative literature review guided the process. The review comprised problem identification, literature search, data evaluation, data analysis and presentation of results. Systematic searches were conducted in the Cinahl, PubMed and Scopus databases.

**Results:**

The search identified 70 articles of which 31, relating to 11 African countries, were used. Of these, 20 were qualitative, 9 were quantitative and 2 were mixed-methods studies. Men’s alienation from health services, and traditional gender norms that discourage fathers’ supportive role during pregnancy were prevalent themes. Financial pressures also dominated fathers’experiences. At the same time, in 18 studies fathers expressed motivation to be involved partners and supportive fathers, despite stigma and exclusion from maternity services.

**Conclusion:**

This integrative review shows that fathers’ experiences of their involvement in their partners’ pregnancy and childbirth across African countries are influenced by multiple factors. While unwelcoming health services, traditional gender norms, and low income are barriers to male involvement, education, younger age, and modern gender norms are associated with greater male involvement.

## Paper Context

- **Main findings:** There is a diversity of experiences among fathers across the African continent, with healthcare system alienation, influential gender norms, and financial pressure being common themes.
- **Added knowledge:** Unwelcoming health services, traditional gender norms, and low income were found to be impediments to male involvement in their partners’ pregnancy and childbirth, while education, younger age, and modern gender norms were associated with greater male involvement.
- **Global health impact for policy and action:** Information and education for men and conducive healthcare environments would enable more positive experiences for men and encourage their greater involvement.

## Background

Masculine roles related to their partners’ pregnancy and childbirth are evolving rapidly in accordance with changing gender norms. Understanding fathers’ experience of involvement in their partners’ pregnancy and childbirth is essential for creating maternal and child healthcare services that are fully inclusive [1]. With economic development and modernization, gender norms tend toward greater equity, especially where social policy supports this [2]. In lower-income countries, where traditional gender norms are more common, male involvement in their partners’ pregnancy and childbirth is more limited [3].

Global research on men’s experiences of being involved with pregnancy and childbirth has found their roles connected to feelings of pride and responsibility [4,5]. Active involvement has shown to enhance connection between partners and family bonding. While some fathers experience stress and overwhelm with aspects of childbirth, being present has increased respect for their partners [6]. Literature indicates that men’s involvement is a key factor in improving maternal and child health outcomes [3,7,8]. Given this, there is a need to encourage active participation in pregnancy and childbirth by male partners, especially in low- and middle-income countries.

Involvement in pregnancy and childbirth of fathers in the African region is still very low [9–12]. Traditional African cultures have predominantly seen and treated maternal and child healthcare as belonging to the female domain, while resource provision belongs to the male domain [11]. Accordingly, fathers have been excluded by health professionals from involvement in antenatal and childbirth care [13]. This is despite their being traditionally the main decision-makers who determine whether women access antenatal and childbirth health services. Men’s roles as providers were further diminished during the COVID-19 pandemic, when economic contraction limited their ability to provide [14–16]. While few African fathers are present during childbirth, a rise in their involvement has been described as a process of social and behavioral change that is necessary to support their partners during pregnancy and childbirth [3,7,13].

This study aims to provide an overview of how fathers in Africa have experienced being involved in their partners’ pregnancies and childbirth. It is anticipated that this knowledge may enable policy makers, healthcare professionals and other stakeholders to adopt practices that are supportive to male involvement, as well as being culturally sensitive. Ultimately, the aim is to support couples as they become parents, thereby promoting optimal family health and wellbeing.

## Methods

### Design

A systematic, integrative literature review inspired by Whittemore and Knafl [17] guided the process. It comprised five key stages: problem identification, literature search, data evaluation, data analysis and presentation of results. This approach was chosen since it allows inclusion of qualitative, quantitative and mixed-methods studies, which is useful in an unexplored research field.

### Literature search

A table listing inclusion and exclusion criteria was developed to guide the review following the PIOS (Participants, Interventions, Outcomes and Study designs) format [18]; for details, see Table 1. The search strategy was developed in October 2021 and updated in January 2024 by two librarians from the University of Gothenburg, Sweden, in collaboration with the authors SCN, AW, HE and MB. Comprehensive systematic literature searches were conducted in three databases: Cinahl, PubMed and Scopus.

**Table 1.** Inclusion and exclusion criteria.

Inclusion criteria	Exclusion criteria
**Participants**	
Cis-gender men from and living in African countries involved in their partner’s pregnancy and childbirth	Queer, non-binary, and transmen; African men with childbearing partners living in non-African countries.
**Intervention** Partners’ pregnancy and childbirth	Surrogate pregnancies, pregnancy loss, abortion, general community perspectives on men’s involvement
**Outcome**	
Cis-gender men’s experience of involvement with partners’ pregnancy and childbirth	Cis-gender men’s experiences of involvement with partners’ pregnancy and childbirth in non-African countries.
**Study design**	
Original research	Systematic literature reviews, commentaries, letters and editorials.
Studies published in English	
Year of publication 2010–2023	

These searches were done using medical subject headings (MeSH) and related abbreviations to focus on relevant papers based on keywords used in the title or abstract. Boolean operators OR and AND were used to retrieve papers relating to the three topics: male partners, African countries, and childbirth. For example, ‘*father*’, OR ‘*men*’, OR ‘*husband*’ AND ‘*Benin*,’ OR ‘*Madagascar*’ AND ‘*pregnancy*,’ OR ‘*birth*’. To maintain the research focus, studies focused on cis-gender men from and living in African countries that explicitly examined men’s involvement in their partner’s pregnancy and childbirth. Studies were limited to original research published in English between 2010 and 2023. The years 2010 to 2023 were chosen because they covered the most recent decade and exclude literature that is largely out of date.

The reference lists from all the articles were screened in their full-text form. After this search, the references were downloaded into the Rayyan web application for systematic reviews, to facilitate the review process [19].

### Study selection

**Figure 1.**
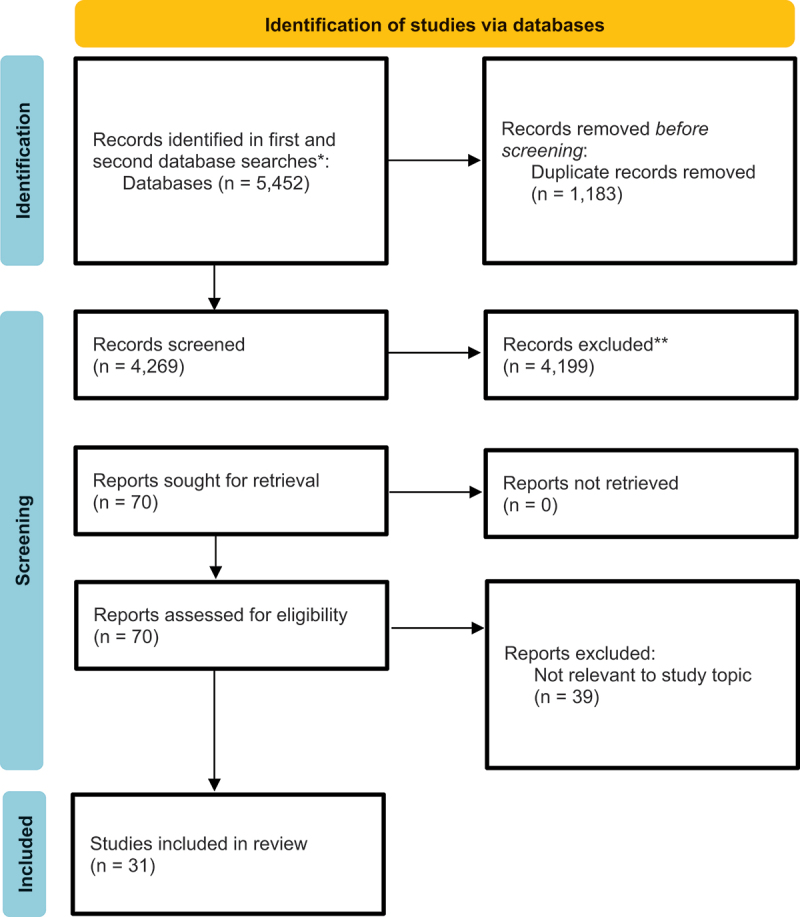
Prisma flow chart.

For the initial screening, all the results from the three searches, comprising 5,452 articles, were imported into the Rayyan web application. Duplication was eliminated, leaving a total of 4,269 titles and abstracts to be screened for inclusion. The four authors (SNC, AW, HE and MB) all independently screened all the titles and abstracts of the 4,269 articles, selecting 70 articles for inclusion in the eligibility assessment. Any disagreements were resolved by consensus. The full texts of the 70 articles were read independently (by SNC & AW), resulting in 31 articles being included. Accordingly, 39 articles were excluded for the reasons presented in Supplementary Table S1. The study selection can be followed in the PRISMA flowchart in Figure 1.

### Data evaluation

Assessment of the quality of the included articles was done using Pluye’s Mixed Methods Appraisal Tool (MMAT). The MMAT is a critical appraisal tool that covers qualitative, quantitative, and mixed-methods study methodologies. For each study type, five criteria are evaluated. The purpose of the first two questions is screening: if both are answered in the affirmative, it is deemed worthwhile to proceed with the remaining questions. Each article was given a score of 1–5 with 5 reflecting that all quality criteria were met (Supplementary Table S2).

All the articles selected for inclusion (*n* = 31) were subjected to rigorous quality appraisal by two of the authors independently (SNC, AW), supervised by HE and MB. ‘High-quality qualitative articles’ were deemed to be those with a well-defined research topic, clear research questions, a high-quality data fit for their intended use, reliability, validity and coherence across data sources, collection, analysis, and interpretation. ‘High-quality quantitative articles’ had a well-defined research topic, appropriate sampling and analysis, and low risk of non-response bias. Mixed-methods articles were assessed for quality of each methodological approach as well as integration of the methods, and discussion of divergences between qualitative and quantitative results. Across articles, the average quality was high, though some articles only met three or four out of the five criteria due to limited availability of information about methods. Table 2 presents the characteristics of the studies included and their quality appraisal.

**Table 2.** Characteristics of studies included and their quality appraisal.

No.	Title Author, year	Method Study design Participants	Objective	Country	Results Quality
1	Husbands’ involvement in birth preparedness and complication readiness in Axum town, Tigray region, Ethiopia.	Quantitative	To assess husbands’ involvement in birth preparedness and complication readiness	Ethiopia	187 (46.9%) men fulfilled five or more parameter variables for husbands’ involvement in birth preparedness and complication readiness; their involvement was found to have been good.
	Baraki et al., 2019 [20]	Cross-sectional			
		406 husbands			Quality: 5
2	Male involvement in the maternal healthcare system: implication towards decreasing the high burden of maternal mortality.	Quantitative	To determine knowledge of obstetric danger signs and involvement among men in a community in northwestern Ethiopia	Ethiopia	412 men (50%) referred to a danger sign that may arise during pregnancy, but none took all the steps of birth preparation during the birth of their most recent child.
	Mersha AG., 2018 [21]	Cross-sectional			
		824 men			Quality: 5
3	Factors affecting men’s involvement in maternity waiting home utilization in North Achefer district, Northwest Ethiopia: A cross sectional study’	Quantitative	Identify factors affecting men’s involvement in maternity waiting home (MWH) utilization	Ethiopia	Men’s involvement in MWH was 55.6%. Age, knowledge about MWH, decision-making power, and receiving counseling about MWH during spousal antenatal care visits had statistically significant associations with men’s involvement in MWH utilization. Quality: 4
	Asmare et al., 2022 [22]	Cross-sectional			
		403 male partners			
4	Extent of male involvement and associated factors in antenatal care service utilization in Bench Sheko zone, Southwest Ethiopia: A community-based cross-sectional study	Quantitative Cross-sectional	Assess extent of male involvement and associated factors in antenatal care service utilization.	Ethiopia	Male involvement in ANC utilization was low (38%). Urban residence, higher level of education, higher income, and higher knowledge about ANC were positively associated with male involvement in ANC utilization. Quality: 5
	Mekonen et al., 2022 [23]	816 men			
5	Factors associated with men’s involvement in antenatal care visits in Asmara, Eritrea: Community-based survey	Quantitative	Estimate the level of male partners’ involvement in ANC visits and identify the associated factors in Asmara	Eritrea	Most study participants believed the pregnancy is a female domain, almost all understood the importance of attending ANC. Religion, education level, and attitude were significantly associated with men’s involvement in ANC.
	Beraki et al., 2023 [49]	Cross-sectional			
		605 men			Quality: 4
6	Social and cultural barriers to husbands’ involvement in maternal health in rural Gambia.	Qualitative 5 FGDs, each group having 8 to 10 participants	To explore underlying social and cultural factors affecting husbands’ involvement in maternal health issues pertaining to pregnancy and delivery in rural Gambia.	Gambia	Rural Gambian men stated that husbands’ involvement in maternal health is highly desirable. Many men did not believe that pregnancy chores warrant their efforts compared with other, competing social responsibilities.
	Lowe et al., 2017	6 IDIs			Quality: 5
7	Inclusion of men in maternal and safe motherhood services in inner-city communities in Ghana: evidence from a descriptive cross-sectional survey.	Quantitative	To quantify men’s inclusion in maternal and safe-motherhood services in Ghanaian inner-city communities, and to assess barriers to men’s involvement	Ghana	114 men (44.5%) accompanied their partners in seeking skilled childbirth services. Men’s involvement was hindered by such barriers as attitudes among health workers, long waiting times and sociocultural beliefs.
	Atuahene et al., 2017 [25]	Cross-sectional 256 men			Quality: 5
8	Husbands’ involvement in antenatal-related care in the Bosomtwe District of Ghana: inquiry into the facilitators and barriers	Qualitative	Exploration of facilitators and barriers to husband’s involvement in antenatal care.	Ghana	Work constraints, traditional gender norms and unwelcoming health facilities were barriers. Facilitators included husbands’ concern for baby, modern gender norms and husbands feeling welcome at facilities due to wive’s receiving preferential treatment for husband being present. Quality: 5
	Morgan et al., 2022 [26]	36 IDIs with male partners, pregnant women, midwives and traditional birth attendants			
9	Barriers to men’s involvement in antenatal and postnatal care in Butula, Western Kenya.	Mixed-methods Cross-sectional 96 men 4 FGDs (8–10 men each)	To explore the barriers to men’s involvement in ANC and PNC in Butula sub-county, Western Kenya	Kenya	Despite the barriers, some men nonetheless accompany their partners to antenatal care. **Time spent at clinics** and lack of privacy there were key facility-based barriers. Financial and **health-facility barriers** hinder men from active involvement in ANC and PNC.
	Ongolly FK, Bukachi SA, 2019 [27]	4 IDIs with key informants			Quality: 3
10	Barriers to male partner accompaniment and participation in maternal and child health care in Thika and Kiambu Level Five Hospital, Kenya	Qualitative	Determine factors influencing male partner accompaniment to maternal and child health department of a teaching hospital.	Kenya	Traditional gender roles and norms, work commitment by men and unfavourable MCH set-up were key barriers identified that hinder men from accompanying their spouses to receive maternal and child health services. Quality: 3
	Okwako et al., 2023 [28]	3 FGDs with nurse-midwives 2 FGDs with male partners			
11	Malawian fathers’ views and experiences of attending the birth of their children: a qualitative study.	Qualitative	To explore the views and experience of men who attended the birth of their children at two private hospitals in an urban setting in southern Malawi	Malawi	Negative experiences were shame and embarrassment, helplessness, and lack of preparedness.
	Kululanga et al., 2012 [29]				
		20 IDIs with men			Quality: 5
12	Factors influencing fathers’ involvement in the care of hospitalized preterm newborns in Balaka, Malawi	Qualitative	Explore factors involved in male involvement in care of hospitalized preterm newborns.	Malawi	The study found that fathers value their involvement in caring for hospitalized preterm newborns, but face barriers related to traditional gender norms, and in some cases hospital and staff related constraints and economic constraints as more than half of participants were unemployed. Quality: 5
	Mhango et al., 2023 [30]	16 IDIs with male partners			
13	Experiences, views, and needs of first-time fathers in pregnancy-related care: a qualitative study in south-east Nigeria.	Qualitative	To explore the experiences and needs of first-time fathers, and how these influenced their involvement during pregnancy and childbirth in Nigeria	Nigeria	Inexperience and perceptions of gender roles greatly influenced the support provided by first-time fathers to their partners.
	Onyeze-Joe et al., 2020 [31]				Two primary needs were identified: to be informed about care in health settings and aware of its costs.
		50 semi-structured interviews with men			Quality: 5
14	Birth Preparedness, Complication Readiness and Fathers’ Participation in Maternity Care in a Northern Nigerian Community.	Mixed-methods	To shed light on men’s participation in maternity care and provide data for development of culturally sensitive strategies for including men in maternal healthcare and delivery in northern Nigeria	Nigeria	Only 128 men (32.1%) had ever accompanied their partners to maternal care facilities. There was very little preparation in terms of saving for emergencies or transport during labour. Young paternal age, formal education, and non-Hausa Fulani ethnicity were independent predictors of male participation in maternity care.
	Iliyasu et al., 2010 [32]	Cross-sectional, 389 men			
		3 IDIs with men (community leaders)			Quality: 3
15	‘You try to play a role in her pregnancy’ – a qualitative study on recent fathers’ perspectives about childbearing and encounter with the maternal health system in Kigali, Rwanda.	Qualitative	To explore recent fathers’ perspectives on maternal care-seeking within the context of Rwanda’s political agenda for gender equality	Rwanda	While the men perceived an obligation for them to accompany their partner on the first ANC visit, they experienced several indications of resistance from the maternal health system to their becoming further engaged.
	Pafs et al., 2016 [33]	32 IDIs with men			Quality: 5
16	A Qualitative Exploration of the Meaning and Understanding of Male Partner Involvement in Pregnancy-Related Care among men in rural South Africa.	Qualitative	To explore the meaning and understanding of male partner involvement as perceived by men visiting primary healthcare clinics in rural communities in Mpumalanga	South Africa	Male involvement was understood as giving instrumental support to female partners through financial help, assisting with physical tasks and providing emotional support. Community attitudes, traditional beliefs and negative experiences in health facilities were barriers.
	Matseke et al., 2017 [10]	6 FGDs involving 53 men			
					Quality: 5
17	Male partners’ views of involvement in maternal healthcare services at Makhado Municipality clinics, Limpopo Province, South Africa: original research.	Qualitative	To determine male partners’ views on their involvement in maternal healthcare services	South Africa	Maternal health issues were viewed as a woman’s domain.
	Nesane et al., 2016 [34]	15 IDIs with men			Quality: 5
18	Exploring the Dilemmas, Challenges, and Opportunities of Adolescent Fatherhood: An Exploratory Case Study	Qualitative	Explore challenges and opportunities of teenage fatherhood in South Africa township	South Africa	Fatherhood affected boys’ education due to the responsibility to provide for their child. Mothers and/or their families sometimes prevented fathers’ access to child if he could not provide financial support. Boys felt a positive responsibility to support and care for their child. They matured.
	Makhavhu et al., 2023 [35]	Semi-structured interviews			
		5 teenage fathers			Quality: 5
19	Navigating relationship dynamics, pregnancy and fatherhood in the Bukhali trial: a qualitative study with men in Soweto, South Africa	Qualitative	Explore individual perceptions around relationship dynamics, partner pregnancy, and fatherhood	South Africa	Most partners not married. Significant concern expressed by fathers about providing for children. Sometimes involvement blocked by partner or her family. Some indication of generational shift in which younger fathers express more motivation to be really involved than earlier generations. Quality: 4
	Draper et al., 2023 [36]	19 IDIs with fathers			
20	Male partners’ experiences of early pregnancy ultrasound scans in Soweto, South Africa: The Healthy Pregnancy, Healthy Baby randomised trial	Quantitative	Explore the experiences and antenatal attachment among male partners who attend early pregnancy ultrasound examinations.	South Africa	While fewer than 50% of women were accompanied by their male partner, the ultrasound overall had a positive effect on men and their thoughts regarding their developing baby. Quality: 4
	Drysdale et al., 2022 [37]	Cross-sectional			
		102 male partners			
21	South African fathers’ experiences with healthcare providers during their partners’ medically high-risk pregnancy and childbirth	Qualitative	Explore fathers’ experiences of interactions with healthcare providers during antenatal stage of partner’s medically high-risk pregnancy.	South Africa	Fathers’ experiences vacillated between healthcare providers being supportive and informative versus them being uncompassionate and not conveying adequate information. Quality: 3
	Richardson et al., 2023 [38]	Semi-structured interviews			
		8 fathers			
22	Caring masculinities? Teenage fathers in South Africa	Qualitative	Explore ways in which a group of teenage fathers talk about their involvement in becoming a teenage father.	South Africa	Father’s roles involved being caring, supporting, and providing materially. Fathers were responsible for paying ‘damages’ for non-marital pregnancy as well as saving for a future marital payment. Participants spoke of ‘real masculinity’ being associated with partnership and nurturing. Quality: 5
	Mvune et al., 2023 [39]	5 FGDs 20 IDIs with teenage fathers			
23	Tanzanian men’s engagement in household chores is associated with improved antenatal care-seeking and maternal health.	Quantitative	This study describes factors associated with men’s involvement in household tasks.	Tanzania	Women from households where men frequently helped were significantly more likely to have taken iron tablets during pregnancy and eaten more than usual. Men had reduced their household workload during their partners’ most recent pregnancies and were more likely to have played with their children in the week prior to the survey.
	Chahalis et al., 2021 [40]	4217 men, 4091 women			Quality: 5
24	Husbands’ experience and perception of supporting their wives during childbirth in Tanzania.	Qualitative	To explore the experience and perceptions of husbands’ support for their wives during pregnancy, labour and delivery in Tanzania	Tanzania	Husbands who supported their partners during pregnancy and delivery saw themselves as modern since, at home, they undertook chores besides their usual tasks to allow their wives adequate time to rest during pregnancy. Poor road infrastructure, hospital ward infrastructure not supporting accommodation for husbands.
	Kashaija et al., 2020 [41]	9 IDIs with husbands			Quality: 5
25	Community perspectives: An exploration of potential barriers to men’s involvement in maternity care in a central Tanzanian community.	Qualitative 32 FGDs, 16 with men/16 with women	To explore community perspectives on potential barriers to men’s involvement in maternity care in central Tanzania	Tanzania	Barriers to men’s involvement in maternity healthcare services are systemic and derived from family, healthcare and culture-specific gender norms for maternity related behaviour and structural constraints on healthcare facilities that inhibit implementation of couple-friendly maternity healthcare services
	Gibore et al., 2020 [42]	34 IDIs with key community members			Quality: 5
26	Perceptions on male involvement in pregnancy and childbirth in Masasi District, Tanzania: a qualitative study.	Qualitative 53 IDIs with key respondents	To explore local perceptions of male involvement in pregnancy and childbirth in Tanzania	Tanzania	Men did not wish to be more actively involved in antenatal care and delivery. Men are breadwinners and their main role is to support their partners financially. Barriers were traditional gender roles at home, fear of HIV testing and unfavourable environment in health facilities.Quality: 5
	Maluka & Peneza, 2018 [43]				
27	Male engagement guidelines in antenatal care: unintended consequences for pregnant women in Tanzania	Qualitative	Examine impact of a policy promoting male engagement in ANC services as part of national plan to eliminate mother-to-child transmission of HIV.	Tanzania	Women whose husbands could not accompany them delayed their first ANC appointments while women who attended with their husbands received preferential treatment and some who presented without their husbands were denied care. Quality: 5
	Osaki et al., 2021 [44]	IDIs with 13 women and 6 male partners			
28	Men in maternal health: an analysis of men’s views and knowledge on, and challenges to, involvement in antenatal care services in a Tanzanian community in Dodoma Region	Quantitative	Determine men’s view, knowledge, and challenges regarding involvement in ANC	Tanzania	Facilitators to involvement were: two-income households, and men’s knowledge of ANC. Barriers were men thinking their only role was to provide money, limited finances esp., where men were only income earner, peer pressure, and feeling that clinic is a woman’s place. Quality: 5
	Gibore & Gesase, 2021 [45]	Cross-sectional			
		966 male partners			
29	The fear of social stigma experienced by men: a barrier to male involvement in antenatal care in Misungwi District, rural Tanzania	Qualitative	Examination of fear of social stigma among men attending ANC with partners	Tanzania	Social stigma related to fear of HIV testing, traditional gender norms, and insecurity about family social and economic status were primary barriers to men participating in ANC. Quality: 5
	Boniphace et al., 2022 [46]	12 IDIs and 5 FGDs with fathers and expectant fathers			
30	An exploratory study of men’s companionship, perceptions and experiences during pregnancy and delivery in Uganda.	Qualitative	To assess companionship during delivery; men’s perception and experience of pregnancy and delivery	Uganda	Men’s involvement in the childbirth process was associated with a more clearly perceived bond with their partners and newborn babies. Their presence helped to promote a calm and successful childbirth process.
	Lwanga et al., 2017 [47]	16 IDIs with men			
					Quality: 5
31	The involvement of men in maternal healthcare: cross-sectional, pilot case studies from Maligita and Kibibi, Uganda.	Qualitative 12 IDIs with men and 23 IDIs with women	To understand, (1) men’s current participation in antenatal, pregnancy and childbirth care, and (2) both men and women’s attitudes towards increased male involvement	Uganda	Men believed that issues related to pregnancy and childbirth were the domain of women. Involvement tended to be confined strictly to traditional gender roles, with men’s main responsibility being provision of funds.
	Singh et al., 2014 [48]	1 FGD with 12 men and 1 FGD with 12 women			Quality: 5

ANC = antenatal care, FGDs = focus-group discussions, HIV = human immunodeficiency virus, IDIs = in-depth interviews.

### Data analysis

The analysis was conducted using the inductive content analysis method, as described by Whittemore and Knafl [17]. First, the articles were read through several times by the authors independently, to gain familiarity with and make sense of the data. In view of the aim of the study, text corresponding to the research questions was systematically annotated.

In subsequent readings data corresponding to the aim was extracted and coded. Similar codes were then compared and clustered in subcategories. Finally, the subcategories were grouped into four main categories. The analytical process was completed by SNC and AW, with repeated discussions until full agreement was reached.

## Findings

In the following section, we describe the fathers’ experiences of involvement in pregnancy and childbirth in Africa.

### Characteristics of studies included

Although the ambition had been to cover all 54 African countries, the final 31 articles only covered 11 countries. The articles’ characteristics are summarised in Table 2. The studies were carried out in Ethiopia [20–23], Eritrea [24], Gambia [24], Ghana [25,26], Kenya [27,28], Malawi [29,30], Nigeria [31,32], Rwanda [33], South Africa [10,34–39], Tanzania [40–46] and Uganda [47,48].

Of the articles reviewed, 20 were qualitative studies: Gambia [24], Ghana [26], Kenya [28], Malawi [29,30], Nigeria [31], Rwanda [33], South Africa [10,34–36,38,39], Tanzania [41–44,46] and Uganda [47,48]. Nine studies reviewed were quantitative: Ethiopia [20–23], Eritrea [49], Ghana [25], South Africa [37], and Tanzania [40]. Two mixed-methods studies were reviewed: Kenya [27] and Nigeria [32].

The synthesis of available research on the fathers’ experience of involvement in their partner’s pregnancy and childbirth in Africa is described in four main categories, each with subcategories (see Table 3).

**Table 3.** Categories and subcategories with descriptions.

Categories	Subcategories and their descriptions
Health services	**Alienation** Feeling alienated from ANC services, and unwelcoming attitudes on the part of health workers. **Rules and physical space** Unfavourable care environment including long waiting time, and fathers not being allowed in exam room or labor ward. **Conditional inclusion** Conversely, feeling included and welcomed, but in some cases primarily for the purpose of 1) enabling preferential treatment for pregnant partner, or 2) receiving an HIV test.
Gender norms	**Dominance of traditional norms** Dominance of traditional gender norms in which pregnancy and childbearing do not require fathers’ involvement, apart from providing financial resources. Social stigma punishing men for playing a supportive role and/or being affectionate with female partner who is pregnant or has children. **Notable shifting norms** In some cases, adoption of more equitable gender norms that elevate and encourage fathers to be supportive, involved partners during pregnancy and childbirth.
Readiness to parent	**Discomfort and challenge** Uncertainty, anxiety, or lack of ability related to normal birth processes, newborn care giving tasks, and taking actions to reduce non-newborn caregiving burdens on partner.
	**Pride and motivation** Personal desire to care for and have an emotionally connected relationship with child.
Logistics	**Pressures of work and need for income** Work responsibilities as a barrier for many fathers in daily wage jobs from attending ANC appointments.Long distances between homes and health facilities as an added impediment to male involvement in maternity care

### Health services

A common theme across articles was fathers feeling alienated from participating in maternal healthcare services. This was due to the structure of care delivery, which included physical spaces, exclusionary rules, and pregnancy information targeting only women. Some articles, however, described scenarios in which antenatal services were highly welcoming to fathers, though these cases were in specific circumstances.

#### Alienation

Twelve articles described a care environment directed toward pregnant and laboring women without attention to or accommodation for male partners [10,25–31,33,38,41,42]. At ANC visits, fathers reported feeling as if providers were only speaking to their pregnant partners, delivering information relevant just to them. They expressed a desire to be able to formally engage with providers, and to receive information targeted to their roles as fathers and partners [38,41]. A study in Malawi found that male participants expressed a desire for information at ANC visits on how they could provide physical support during labor. When they tried to seek such support for their laboring partners, they felt dismissed [29]. A study in South Africa pointed out that fathers felt out of place when they accompanied their partner to health facilities [10]. Another in Rwanda reported that some fathers wanted to attend their children’s births, but were prohibited from doing so by their wives [33]. These experiences, reported across seven countries, indicate that men feeling excluded from involvement in their partner’s maternity care is a significant aspect of fathers’ experiences of pregnancy and childbirth.

#### Rules and physical space

Thirteen studies revealed barriers to fathers’ involvement related to infrastructure, and both formal and social rules governing access to different areas within hospitals [10,20,24–26,32–34,38,41–43,46]. Many health facilities lacked space to accommodate couples. Frequently, fathers wanting to be present for their child’s birth were not able to due to rules prohibiting them from entering labor wards. This was specifically mentioned in studies in Ethiopia [20], Gambia [24], Nigeria [32], Rwanda [33], and Tanzania [41]. Lack of privacy for other women being examined or performing kangaroo mother care was a social barrier to fathers’ entry into those spaces as well [27,30]. Other physical space-related barriers were that there were no specific areas designated for men, making it difficult when fathers had to wait for long periods – with their partner until she was seen and then alone until her visit was completed [10,20,25,28]. Such concerns discouraged fathers’ involvement.

#### Conditional inclusion

A minority of studies reported fathers feeling welcomed, even strongly encouraged, to participate in maternity care services. Two in which fathers felt included were in specific circumstances of high-risk pregnancy and newborns in neonatal intensive care [30,38]. In addition, multiple studies reported that women who attended ANC visits with their male partners were given priority and had a shorter wait time. In some cases, men waited in line to hold a spot for their partner and then left for work when she arrived [26]. Studies also reported fathers’ concerns around being required to take an HIV test if they accompany their partner to ANC services. One study in Tanzania reported participants feeling excessive pressure to attend ANC visits, and that providers seemed to value them taking an HIV test over their engagement with their partner’s pregnancy care [44].

### Gender norms

The literature reflected a dominance of traditional gender norms relegating pregnancy and childbirth to the women’s domain. However, this was not universal, and studies reflected a diversity of views. Fathers ubiquitously described their roles as financial providers. Some also described being involved in their partner’s antenatal appointments, providing emotional support, being present and caring fathers, and taking over childcare and household chores while their partner recovered from childbirth. In a few studies, fathers reported facing barriers to playing these roles, despite their wanting and/or trying to.

#### Dominance of traditional norms

Traditional views on gender norms related to pregnancy and childbirth were expressed in 23 studies. Though a common theme mentioned by study participants in all 11 countries, most studies reflected a mix of both traditional and more egalitarian views. Studies reporting a dominance of traditional gender norms largely sampled low-income fathers from rural or otherwise marginalized communities. For example, studies from Kenya [27,28], South Africa [34] and Tanzania [42,45] in which men described not being involved in their partners’ pregnancy and childbirth experiences (apart from providing financial resources), sampled primarily casual laborers, subsistence farmers, and/or unemployed fathers.

A related theme fathers frequently shared was they would be treated poorly by their peers if they displayed involvement and supportive gestures toward their partners. Stigma directed at men for playing a supportive role during their partner’s pregnancy was discussed in six studies [10,25,28,31,41,45], leading some men to hide such behaviors and only display support while out of public view. In Nigeria for instance, members of the historically marginalized Igbo tribe reported displaying traditional norms in public and behaving in a more supportive manner in their homes [31]. Other articles mentioned fathers’ concern about being judged or ridiculed by other men for accompanying their partner to ANC visits or providing her with extra care during pregnancy. Male involvement behaviors in these studies were described as shameful as they would imply that a man’s partner controls him [10,28,45].

On the contrary, studies reported fathers wanting to be present and involved during their child’s birth and infancy, but being prevented from doing so. This was mentioned in studies looking at adolescent fathers who wished to build a relationship with their child as well as provide financial support [35,36,39]. For many adolescent fathers, having a child limited their education as generating income became a higher priority. At the same time, when couples were not married, mothers’ families would sometimes block fathers’ access to their children, demanding financial commitment or payment first. In other cases, social or cultural norms (e.g. the pregnant woman not wanting her partner present, or a woman leaving her partner’s home and living for a period with her parents) dictated fathers’ exclusion from childbirth [10,32,33].

#### Notable shifting norms

Overall, however, a wide diversity of views and experiences was conveyed in the research. Eagerness and responsibility to care for their partner and be involved in pregnancy and childbirth was conveyed by men in 18 studies [10,21–23,26,29–36,39–41,47,49]. Multiple studies presented data reflecting both traditional and more egalitarian views coming from different sample cross-sections. Some studies inferred influential factors, concluding that fathers with higher levels of education, who lived in more urban areas, had more knowledge about maternity services, and/or were from younger age cohorts were more likely to be involved partners [21–23,26,29,35,36,39–41,47,49]. Men from these groups more than others expressed the desire to be active participants in the pregnancy and childbirth process. Some variation was apparent in that, in one study, fathers’ involvement was associated with men being older [22]. It was also notable that education was a stronger driver of male involvement than income, profession, or socioeconomic status. Three studies, for instance, conveyed sentiments of eagerness and responsibility for involvement from low-income fathers [33,39,40].

### Readiness to parent

Another prevalent theme across studies was fathers feeling unprepared to parent. Feelings of uncertainty and anxiety related to normal birth processes and newborn caregiving tasks were shared by fathers. Some conveyed feelings of discomfort connected to carrying out tasks at home typically performed by their partner. Conversely, in a handful of studies, fathers described pride, motivation and a strong sense of responsibility for caring for their partner and bonding with their child.

#### Discomfort and challenge

Men reported experiencing stress in hospital settings related to lack of knowledge, their partners’ labor pain, and carrying out newborn care tasks as instructed by nurses. They described feeling fearful not knowing what to expect, and powerless to reduce their partners’ pain [47]. One study described fathers’ overwhelm at normal bleeding during labor and discomfort with seeing vaginal exams take place. This same study, however, also reported fathers appreciating being able to negotiate services to relieve their partners’ pain [29]. A study on Malawian fathers’ involvement with their preterm newborns found that fathers expressed fear at carrying out newborn care tasks incorrectly, or potentially hurting them when holding or bathing them due to their small size and fragility [30]. As newborns grew, though, they overcame their fear, and performed tasks such as skin-to-skin and bathing, despite those tasks initially feeling challenging.

#### Pride and motivation

As mentioned, multiple studies conveyed fathers’ personal desire to care for and have an emotionally connected relationship with their child. One study of Ugandan couples found that fathers who played a supportive role during labor (e.g. being a companion, providing massage, assisting with movement, bringing tea, etc.) felt more strongly bonded to both their partner and child [47]. This sentiment was also expressed in a study of men who attended their children’s birth in two private hospitals in southern Malawi [29]. A study in Tanzania found that men were motivated by love, duty, and their partner’s and child’s wellbeing to provide care alongside their partner during her labor [41]. Fathers in this study also described carrying out cooking and cleaning at home to enable their partners to rest and recover from childbirth. Another study in Tanzania described men being more involved in household chores and caring for their older children during their partners’ pregnancies [40]. Three studies of fathers’ experiences in South Africa shared their positive emotions regarding feeling attached and building love and connection with their children [35–37].

### Logistics

Certain barriers identified were related to logistics. Men’s work and income pressures were a frequently mentioned barrier to them accompanying their partners to health services. Long distances to facilities were a related factor.

#### Pressures of work and need for income

Financial pressure was cited by fathers in seven studies as a barrier to their participation in ANC services [28,30,35,36,41,45,46]. The pressure of needing to provide for their families and the possibility of a lost day of income were major elements of fathers’ experience when determining their level of involvement in their partner’s pregnancy and childbirth process. One study reported the responsibility to provide financially as the most challenging aspect of fatherhood [36]. Fathers who worked as casual laborers or small business owners reported a direct relationship between the time required to support their partners and their daily cash flow [41]. In a study carried out in Tanzania, participants from one ethnic group shared that fathers who could not afford nice clothing avoided accompanying their partner to facility visits as they felt shame due to their lack of material resources [46]. Another study in Tanzania found that in dual-income households, fathers were more likely to attend ANC services [45]. As mentioned, adolescent fathers experienced unique challenges related to income [35]. Providing financially often meant sacrificing their education. In some cases, adolescent fathers’ access to their offspring was blocked due to their not providing enough funds. Men’s workplace being a long distance from health facilities was also described by multiple fathers as a barrier to attending ANC visits [28,30,34].

## Discussion

This integrative systematic review identified how fathers have experienced being involved in their partners’ pregnancies and childbirths in 11 African countries. Fathers reported alienation and exclusion from health facilities, and in some cases from their children. Instigated primarily by health facility staff and existing rules, some men were also excluded by their partners and/or their families. Another factor that dissuaded fathers from being involved, supportive partners and fathers was social stigma from peers shown through teasing about men’s partners having power and control over them. This was more common among lower income fathers and influenced by traditional gender norms. The results, however, reflect a variety of views and experiences, including those of fathers’ with a high interest in being involved. Factors associated with fathers’ involvement were higher levels of knowledge about pregnancy and childbirth, more education, younger age, and modern gender norms. Men’s expression of responsibility and/or stress around fulfilling the financial requirements of childbirth was universally shared across paper in this review. As the most common experience fathers have, the pressure to be present at work instead of with their partner or child was lighter for more affluent fathers and for those whose partner worked. Despite high motivation and less social stigma around emotionally connected fatherhood, adolescent and unmarried fathers face unique barriers to involvement. Becoming a father limited their educational opportunities and it was not uncommon that mothers’ families made seeing their children contingent on young fathers making financial payments.

Some of these same topics have been noted about the experiences of fathers in and outside the African context, indicating they may be more global than continent specific. For instance, fathers’ unique information needs related to pregnancy and childbirth have been reported on in individual country studies and systematic reviews globally. Articles describe fathers in LMICs and high-income countries as having information needs that would ideally be met with customized information provided to them directly during the pregnancy period [3,50,51]. Calls for accurate information for fathers about childbirth, ways to be supportive during pregnancy, and newborn care consider it a catalyst for strengthening male partners in their transition to fatherhood [4,52,53]. An integrative review that primarily included studies carried out in Europe found that an estimated 13% of men experience debilitating fear during their partner’s pregnancy which would best be met through knowledge-focused antenatal sessions aimed at building fathers’ childbirth self-efficacy [54]. Other studies that look at stress that is unique to fathers describe them feeling it necessary to hide their stress when their partner gives birth to perform the role of a strong supportive partner [55,56]. The findings in these global research articles echo the experiences identified in this review of African fathers feeling uncertain about how to provide effective partner support and newborn care, and also wishing that antenatal care services had components that were designed for fathers to support their new roles.

While unwelcoming health facilities and long waiting times are not discussed as prolifically in the global literature, ample evidence indicates that fathers experience similar types of alienation and exclusion. For instance, Baldwin et al. found in a systematic review of studies from Australia, Canada, Japan, Singapore, Sweden, Taiwan, the UK, and the US that health providers tended to not involve fathers, or treat them as equal partners [4]. Corroborating this, Jackson et al. (2023) reported in a scoping review on men’s experiences of their partners’ high-risk pregnancies looking at research from Germany, Malawi, Sweden, Taiwan, Thailand, Uganda, the UK and the US that fathers felt neglected by providers. This led to their feeling anxious, powerless, and unprepared to address challenges [55]. In a qualitative study of Australian fathers’ experiences with antenatal care, a predominant theme that arose was fathers feeling sidelined within health service settings, almost ‘invisible’ [57]. A study in Sweden found that policies that restricted partners from entering postnatal care wards during the COVID-19 pandemic resulted in dissatisfaction with care provided and fathers feeling excluded as partners and parents [58]. Despite the commonality of fathers’ exclusion across countries, greater structural barriers to fathers’ involvement are apparent in the research focused on LMICs, where 33% to 95% of male partners face restrictions in being present in the room when their partner gives birth [59]. Notably, a study among policymakers representing five Pacific islands found that facility layouts were not ‘male friendly’ with fathers waiting outside while their partners were seen inside. This was largely due to lack of adequate space for private consultations and limited staff time to address and accommodate fathers [60].

In contrast to fathers’ common experience of alienation, this review also found positive experiences of men whose partners had high-risk pregnancies, or whose newborns required time in intensive care. With some exceptions, the global literature mirrors this by indicating that fathers generally feel welcomed and included, but have specific information needs and anxieties that require attention [5,61,62]. In contrast, a study of fathers whose wives experienced childbirth complications in Mulago Hospital in Uganda reported strong feelings of lack of inclusion for them as partners, largely due to lack of space, restrictions in their entering delivery rooms, and their general treatment as visitors rather than partners and carers [63]. Yet, all these findings further highlight that fathers’ experiences and needs are unique and recommend that programs be designed to cater to fathers as individuals with information and support needs specific to their roles as fathers, separate from mothers.

The profound financial pressure this review revealed as experienced by fathers in the African literature, described by some as ‘the greatest challenge’ in their experience, is not reflected in the same way in the global literature. It still appears, but not as prominently. Much of the global literature discusses this topic as part of the picture for male partners – an important stressor among many [4]. Fathers are assumed and expected to be breadwinners by health facility staff, and describe work being a barrier to their involvement in their partner’s pregnancy and childbirth [60,64,65]. For men working as casual laborers or in the informal economy, missing work to participate in their partners’ pregnancy and childbirth care means less money to put food on the table. Higher-income fathers have more freedom to participate, but face social pressures within their workplaces to be engaged employees more so than engaged fathers [66]. One study of fathers’ health information needs among a small sample of men in the US found notable differences in the information needs of lower- compared to higher-income fathers [50]. Lower-income fathers expressed greater interest in topics such as adoption, child support, community resources, jobs, where to find inexpensive cribs, helping a partner during pregnancy, and being a responsible father. Higher-income fathers reported greater comparative interest in caregiving for their partner and child during and post childbirth, signs of an abnormal pregnancy, and depression.

Unique financial pressure among teen fathers also stood out in this review. This was most pronounced in the studies on young South African fathers facing intense pressure to provide funds in order to see their children. The global research on adolescent fatherhood found differences in the experiences of young fathers across countries and regions. Two studies of teenage fathers in Thailand found themes of young fathers taking on roles of breadwinners and involved parents that corresponded with the three studies of young fathers from South Africa [67,68]. However, all Thai study participants were living with their child’s mother (many in an extended family situation), whereas most young African fathers were not cohabiting with their partner. Secondly, greater support – financial as well as emotional, physical, material, and informational – from families was documented in the Thai studies, which contrasted with the animosity and financial demands reflected in the South African studies. A study in the UK found that young fathers are increasingly living apart from their children’s mother, however, they tend to be engaged in providing both financial and caregiving support despite living apart [69]. Another study that looked at adolescent fathers in Ethiopia, India, Peru and Vietnam found that adolescent fathers were more likely to leave school early, have physical and mental health issues and spend less time on leisure activities than nonfather adolescents [70].

Overall, this review found as many similarities as there were differences among African fathers in Africa, and among African fathers and fathers globally. The results point to education, particularly education designed for and delivered directly to fathers, on pregnancy, childbirth, and parenting, as a primary solution area for supporting fathers in their transition to engaged fatherhood. In addition to education, supporting fathers in many African countries will require modifying health facility layouts to incorporate space considerations for couples (in particular privacy for women), revising policies to promote father involvement, and training providers on inclusive communication [71]. Issues around stigma toward men for being involved and supportive during their partners’ pregnancy, and fathers’ roles as providers – in particular the extreme pressure to provide financially in some cases – are larger societal dimensions of the issue that are outside the scope of a single approach. They will both influence change in the other areas and be an outcome of that change. Future research should include rigorous studies on barriers and facilitators to engaged fatherhood in African countries not yet reflected in the literature. Programs and associated evaluations should invest in father-focused education programs, and on shifting health service delivery approaches toward inclusive design. Doing so will not just improve men’s experiences and roles as supportive partners, but will also contribute to improved maternal, child and family health [3,7,72].

## Methodological considerations

The systematic integrative literature review covered articles from only 11 African countries, limiting its generalizability across the diverse African continent. Despite this, the high quality of the selected studies supports the findings. Studies scoring below 5 on the MMAT quality appraisal tool lacked adequate information but did not impact the overall categories, which were supported by multiple studies. The integrative design enhances the transferability of findings [17] across different African contexts, offering a comprehensive understanding applicable to broader settings. However, transferability is influenced by study heterogeneity and potential overgeneralization [73], making it crucial to account for differences in study design, population, and context to enhance relevance and applicability.

## Conclusion

As male involvement in pregnancy and childbirth has a positive impact on maternal health, understanding African men’s experiences of involvement is important for improving maternal health. We found commonalities as well as diversity in men’s experiences. Health system alienation, traditional gender norms, and low income were common challenges. A universal part of men’s experience is the need to provide financially, and this at times is a barrier. Fathers’ proactive desire to be involved, influenced by education (both general and on maternal health specifically) and modern gender norms, was conveyed across multiple studies. Greater affluence and the associated ability to be away from work enabled men’s involvement, but supportive, caring involvement did not depend on it. Information and education for men and conducive healthcare environments would enable more positive experiences for men and encourage their greater involvement.

## Supplementary Material

Supplemental table 2_quality appraisal_MMAT.xlsx
